# Psychometric properties of the Hospital Survey on Patient Safety Culture for hospital management (HSOPS_M)

**DOI:** 10.1186/1472-6963-11-165

**Published:** 2011-07-11

**Authors:** Antje Hammer, Nicole Ernstmann, Oliver Ommen, Markus Wirtz, Tanja Manser, Yvonne Pfeiffer, Holger Pfaff

**Affiliations:** 1 Institute for Medical Sociology, Health Services Research and Rehabilitation Science, Faculty of Human Science and Faculty of Medicine, University of Cologne; Cologne; Germany; 2 Institute for Psychology, University of Education Freiburg; Freiburg; Germany; 3 University of Fribourg, Department of Psychology; Fribourg; Switzerland; 4 ETH Zurich - Center for Organizational and Occupational Sciences; Zurich; Switzerland

## Abstract

**Background:**

From a management perspective, it is necessary to examine how a hospital's top management assess the patient safety culture in their organisation. This study examines whether the Hospital Survey on Patient Safety Culture for hospital management (HSOPS_M) has the same psychometric properties as the HSOPS for hospital employees does.

**Methods:**

In 2008, a questionnaire survey including the HSOPS_M was conducted with 1,224 medical directors from German hospitals. When assessing the psychometric properties, we performed a confirmatory factor analysis (CFA). Additionally, we proved construct validity and internal consistency.

**Results:**

A total of 551 medical directors returned the questionnaire. The results of the CFA suggested a satisfactory global data fit. The indices of local fit indicated a good, but not satisfactory convergent validity. Analyses of construct validity indicated that not all safety culture dimensions were readily distinguishable. However, Cronbach's alpha indicated that the dimensions had an acceptable level of reliability.

**Conclusion:**

The analyses of the psychometric properties of the HSOPS_M resulted in reasonably good levels of property values. Although the set of dimensions within the HSOPS_M needs further scale refinement, the questionnaire covers a broad range of sub-dimensions and supplies important information on safety culture. The HSOPS_M, therefore, is eligible to measure safety culture from the hospital management's points of view and could be used in nationwide hospital surveys to make inter-organisational comparisons.

## Background

Safety culture is an aspect of organisational culture that relies on safety issues in organisations. Guldenmund characterised organisational culture as a relatively stable, multidimensional construct that depends on shared values and norms in the work environment [1]. These values and norms affect the attitudes, perceptions and behaviour of all organisational members. Safety culture, as a part of organisational culture, therefore, has a fundamental impact on safety behaviour and, in turn, on safety in organisations as a whole. It is a multidimensional phenomenon that is defined here as a common stock in knowledge, values and symbols about patients' safety. Organisations with a positive safety culture are characterised by open communications based on a common foundation of values and trust, as well as shared perceptions and mutual support among individual organisational members [2,3].

Measuring safety culture in health care received increased attention at the end of the 1990s. This was contributed to by the publication of the report *To err is human *by the Institute of Medicine (IOM) [4]. Early measures of safety culture in health care were adapted from those used in industrial sectors in the late 1990s [5]. Since then, a large number of surveys have been published regarding safety culture in health care settings [6-8].

Nonetheless, safety culture is still a major issue in health care research. This is reinforced by the increasing external pressure on health care organisations to provide safe and high quality health care. Prior research has concluded that the attitudes, perceptions, expectations and actions of hospitals' top management regarding patient safety is an essential dimension of safety culture [5,7-12]. Organisational improvement towards safety culture is largely based on the commitment of the hospital management, whose members, such as medical directors, are essential decision makers. The hospital management is responsible for the establishment of policies and procedures on quality improvement and hospital safety culture [13]. From the management perspective, it is necessary to examine how they assess safety culture in their own organisations. This point of view on hospital safety culture is important for their decisions. In addition, Rousseau [14] stated that the use of key informants is common practice in research into organisational culture. Key informants, such as members of the hospital management, are presumed to have a comprehensive knowledge about their organisations. However, most safety culture surveys measure safety culture from the frontline staff's points of view.

Although the commitment and perceptions of hospital managers are expected to be important for patient safety, there exists no tool for measuring safety culture from the hospital management's points of view. Therefore, the well-known Hospital Survey on Patient Safety Culture (HSOPS) [15] was adapted to assess the safety culture perceptions of hospital managers, such as medical directors, in German hospitals (HSOPS_M). Thus, the purpose of this study was to test (1) whether the HSOPS_M could be used in a management survey and (2) if the psychometric properties of the HSOPS_M are comparable to those of the HSOPS for hospital employees.

## Methods

### Study design and population

The following analyses are based on data of the project *Effects of Hospital Ownership Structures on Quality of Health Care *(HOSQua) funded by the German Medical Association. This study has been approved by the Research Ethics Board at the University of Cologne.

Data were gathered between April and October 2008 within a cross-sectional, retrospective postal survey. The questionnaires were distributed to 1,224 medical directors from all German hospitals that fulfilled the following criteria: at least one internal medicine and one surgery unit. In order to increase the response rate we examined the classic total design method by using timed reminder and follow-up mailings (including the questionnaire again) [16,17].

### Measure

The HSOPS has been used worldwide in more than 30 countries. As far as is known, this survey for hospital employees has been translated and adapted for use in 14 European countries (Belgium, Denmark, France, Ireland, Italy, the Netherlands, Norway, Portugal, Scotland, Spain, Sweden, Switzerland, Turkey and the United Kingdom). To measure safety culture from the hospital management's points of view in German hospitals, we adapted the Swiss-German version [18] of the HSOPS to assess medical directors' views of German hospitals. The final version for the hospital management, especially the medical directors (HSOPS_M), slightly differs from the original HSOPS (e.g. we excluded the individual item outcome measure *Number of events reported*; the management version only uses the term *staff *instead of *people*). It consisted of 43 items: 10 safety culture dimensions and two outcome dimensions, as well as the individual item outcome measure *Patient safety grade*. The questionnaire scale items are listed in Table 1. Most items of these dimensions are rated on a five-point Likert scale ranging from *I strongly disagree *(1) to *I strongly agree *(5). Some items are rated on a five-point frequency scale from *Never *(1) to *Always *(5). The HSOPS_M questionnaire can be downloaded as additional file 1: Hospital Survey on Patient Safety Culture for hospital management (HSOPS_M). A German version of HSOPS_M is available on request.

**Table 1:** Questionnaire scale items

Dimension	Item	
***Safety Culture Dimensions***		

Hospital management support	F1	Hospital management provides a work climate that promotes patient safety.
for patient safety	F8	The actions of hospital management show that patient safety is a top priority.
	F9r	Hospital management seem to be interested in patient safety only after an adverse event happens.

Supervisor/manager expectations/actions	B1	Supervisors/managers say a good word when they see that a job has been done according to established procedures (standards and guidelines).*
	B2	Supervisors/managers seriously consider staff suggestions for improving patient safety.
	B3r	Whenever pressure builds up, supervisors/managers want staff to work faster, even if it means taking shortcuts or skipping steps.
	B4r	Supervisors/managers overlook patient safety problems that happen over and over.

Teamwork across hospital	F2r	Hospital units do not coordinate well with each other.
units	F4	There is good cooperation among hospital units that need to work together.
	F6r	It is often unpleasant for staff from one hospital unit to work with staff from other hospital units.
	F10	Hospital units work well together to provide the best care for patients.

Teamwork within units	A1	Staff support one another within the units.
	A3	When a lot of work needs to be done quickly, staff within the units work together as a team to get the work done.
	A4	Staff within the units treat each other with respect.
	A11	When one area within a unit gets really busy, others help out.

Communication openness	C2	Staff within units will freely speak up if they see something that may negatively affect patient care.
	C4	Staff within units feel free to question the decisions or actions of those with more authority.
	C6r	Staff within units are afraid to ask questions when something does not seem right.

Hospital handoffs and	F3r	Things "fall between the cracks" when transferring patients from one unit to another.
transitions	F5r	Important patient care information is often lost during shift changes within the hospital units.
	F7r	Problems often occur during the exchange of information across hospital units.
	F11r	Shift changes are problematic for patients within the hospital units.

Nonpunitive response to error	A8r	Staff within the individual units feel like their mistakes are held against them.
	A12r	When an event (e.g., mistake) is reported, it feels like the person is being written up, not the problem.*
	A16r	Staff worry that mistakes they make are kept in their personnel file.

Feedback and communication about errors	C1	Staff within units are given feedback about changes put into place based on events reported (e.g., mistakes).*
	C3	Staff within units are informed about events (e.g., errors) that happen in their units.*
	C5	Staff within units discuss ways to prevent an event (e.g., error) from happening again.*

Staffing	A2	Units within this hospital have enough staff to handle the workload.
	A5r	Unit staff work longer hours than is best for patient care.
	A7r	The units use more agency/temporary staff than is best for patient care.
	A14r	Staff within units work in "crisis mode" trying to do too much, too quickly.

Organizational learning	A6	Staff within the units are actively doing things to improve patient safety.
	A9	Mistakes have led to positive changes within the hospital units.
	A13	After changes have been made to improve patient safety within the units, their effectiveness is evaluated by the staff.

***Outcome Dimensions***		

Overall perceptions of safety	A10r	It is just by chance that more serious mistakes don't happen within the units.

	A15	Patient safety is never sacrificed to get more work done.
	A17r	We have patient safety problems within the units.
	A18	Unit procedures and systems are good at preventing errors from happening.

Frequency of event reporting	D1r	When an event (e.g., error) occurs that is caught and corrected before affecting the patient, how often is this reported?*
	D2r	When an event (e.g., error) occurs that poses no potential harm to the patient, how often is this reported?*
	D3r	When an event (e.g., error) occurs that could harm the patient, but does not, how often is this reported?*

*NOTE: *Items marked with * include very special adoptions for a survey in Germany. For these items, additional translations closer to the original HSOPS version are available.

### Data analyses

Before starting the in-depth analysis, respondents with missing values of > 30% in scale items were excluded because of the limited data quality. Afterwards, missing values were replaced by a multiple imputation based on expectation maximization (EM) algorithm with the statistical software NORM 2.03 [19]. Further analyses were started after a necessary reverse coding of negatively worded items.

Within the pre-analyses, we calculated the Kaiser-Meyer-Olkin (KMO) and Measure of Sample Adequacy (MSA) coefficients. The value of the KMO coefficient indicates whether the sample of items is adequate for a factor analysis or not, whereas the MSA coefficient proves whether a single item is suitable for a factor analysis or not. For both, KMO coefficient and MSA coefficient values of > .60 imply a good applicability and values of > .90 imply a perfect applicability [20]. Finally, we performed Bartlett's test. A high significant *p*-value (*p *< .001) indicates an appropriate dataset for factor analysis.

Using the maximum likelihood method, we performed a ***confirmatory factor analysis ***(CFA) [21] to check whether the theoretical and empirical developed factor structure of the original version for hospital employees fits to the data of the German version for the hospital management. The appropriateness of the CFA model was assessed by measures of global and local fits [21,22]. To evaluate the global fit of the 12-factor model we assessed the goodness-of-fit with the Chi-squared values, which indicate the difference between the observed and the expected covariance matrices [21,22]. To reduce the sensitivity of the Chi-squared value to the sample size we computed a normed Chi-squared value (Chi^2^/df) using the recommended cut-off value of ≤ 2.5 [23]. In addition, the following incremental and descriptive measures of model fit were calculated: (1) Comparative Fit Index (CFI); (2) Tucker-Lewis Index (TLI); (3) Root Mean Square Error of Approximation (RMSEA); and (4) Standardised Root Mean Residual (SRMR). Using recommended criteria for a sample size N > 250 and a number of observed variables m > 30, we determined the cut-off values of ≥ .90 for CFI and TLI, ≤ .07 for RMSEA and ≤ .08 for SRMR [22]. Furthermore, the local fit of the items of the proposed factor structure were estimated with the following criteria and cut-off values: indicator reliability (≥ .30), factor reliability (≥ .60) and Average Variance Extracted (AVE; ≥ .50) [24]. These local fit indicators assess the degree to which the instrument is reliable and valid.

In a second step, the ***construct validity ***was tested by calculating the Fornell-Larcker Ratio (FLR) [25-27]. According to Fornell and Larcker [26], discriminant validity is given when the AVE of a factor is greater than the highest squared inter-correlation with any other factor of the model. Values of < 1 indicate that constructs within the model are sufficiently distinguishable. Furthermore, we calculated Pearson's correlation coefficients for all 12 safety culture dimensions after calculating a composite score for each dimension. According to Campbell and Fiske [28], we determined a cut-off value of ≥ .70. Higher values indicate that the dimensions measure the same concepts. Pearson's correlation values of < .20 would indicate a poor relationship between two safety culture dimensions [29]. Despite all 12 dimensions measure safety culture, we assumed that sufficient inter-correlations would be reflected by moderate Pearson's correlation coefficient values. Additionally, we calculated correlations between the 12 safety culture dimensions and the individual item outcome measure *Patient safety grad*e. We expected significant positive correlations.

Finally, the ***internal consistency ***was measured by using Cronbach's alpha for each of the 12 dimensions. Cronbach's alpha is a measure of how strongly items are correlated [30]. A value of zero indicates no correlation between the items, whereas a value of one indicates a perfect correlation. If items are related too closely, the information of the items is redundant. Therefore, a good value of Cronbach's alpha is between .70 and .90 [20,21,30]. The reliability of the scales were compared to the results of the original HSOPS by Sorra and Nieva, who defined the acceptable level of Cronbach's alpha as ≥ .60 [15].

All statistical analyses were performed using the statistic software SPSS 18.0 and AMOS 18.0.

## Results

### Sample characteristics

The overall response rate was 45% (551 out of 1,224 questionnaires). Of those, four respondents were excluded because of limited data quality (missing values of > 30% in scale items). Finally, 547 questionnaires were included in the analysis. For these, we observed a mean average of 0.32% missing values in the items of the scales. The missing values were imputed applying the EM algorithm [19]. Descriptive statistics, including means and standard deviations for all items within the scales, are presented in Table 2.

**Table 2:** Descriptive statistics of the scales and items included in the CFA

Dimension	Item	Mean per item	Standard deviation per item	Overall mean per scale	Overall standard deviation per scale
***Safety Culture Dimensions***					

Hospital management support for patient safety	F1	3.69	.766	3.73	0.81
	F8	3.61	.985		
	F9	3.90	.968		

Supervisor/manager expectations/actions	B1	3.48	.706	3.68	0.52
	B2	3.91	.616		
	B3	3.44	.824		
	B4	3.91	.721		

Teamwork across hospital units	F2	3.57	.832	3.80	0.59
	F4	3.84	.689		
	F6	3.89	.782		
	F10	3.88	.710		

Teamwork within units	A1	3.80	.663	3.56	0.56
	A3	3.57	.770		
	A4	3.58	.690		
	A11	3.30	.799		

Communication openness	C2	3.82	.672	3.68	0.55
	C4	3.26	.720		
	C6	3.95	.775		

Hospital handoffs and transitions	F3	3.49	.813	3.56	0.68
	F5	3.44	.878		
	F7	3.66	.761		
	F11	3.64	.875		

Nonpunitive response to error	A8	3.71	.809	3.87	0.69
	A12	3.96	.869		
	A16	3.93	.902		

Feedback and communication about error	C1	3.80	.820	3.80	0.66
	C3	3.81	.802		
	C5	3.78	.739		

Staffing	A2	2.70	.901	3.19	0.71
	A5	3.19	1.058		
	A7	3.96	.891		
	A14	2.90	.930		

Organizational learning	A6	3.83	.587	3.64	0.53
	A9	3.61	.749		
	A13	3.49	.773		

***Outcome Dimensions***					

Overall perceptions of safety	A10	3.93	.897	3.62	0.63
	A15	3.22	.969		
	A17	3.87	.782		
	A18	3.44	.737		

Frequency of event reporting	D1	3.38	.934	3.33	0.78
	D2	3.07	.896		
	D3	3.54	.825		

The value of the KMO coefficient was .947. This indicated that the patterns of correlations are very compact and a factor analysis is appropriate for our data [20]. The values of the MSA coefficients ranged between .87 (A5) and .98 (C5). Except for two items (A5 and D2), all items reached a superior value of > .90. Therefore, both the KMO test and the MSA test indicated that the data fit the criteria for a factor analysis [20]. Finally, the *p*-value of < .001 within Bartlett's test indicated an appropriate data structure for applying factor analysis.

### Confirmatory factor analysis

The results of the CFA model indicated a satisfactory global data fit: Chi^2 ^= 1,632.71; df = 753; *p *< .000; Chi^2^/df = 2.168. According to the criterion by Hair et al. [22], of the more than 30 observed items and the minimum of 250 observations the model exhibited an acceptable-to-good global data fit (Table 3).

**Table 3:** Model fits of the 12 HSOPS_M dimensions

Model Fit Index	Criterion (N > 250 and m ≥ 30)	Fit index German sample
Chi^2^		1632.708
df		753
*p*	< .05	.000
Chi^2^/df	< 2.5	2.168
CFI	> 0.90	.916
TLI	> 0.90	.904
RMSEA	≤ 0.07	.046
SRMR	< 0.08	.048

*NOTE: *For thresholds of acceptable fit see Hair et al. [22] and Bollen [23]

Furthermore, two out of three indices of local fit, as presented in Table 4 indicated a good convergent validity: except for the dimension *Supervisor/manager expectations/actions*, no scale included more than one item with an indicator reliability ≤ .30. Additionally, the factor reliabilities exceeded the recommended critical values of ≥ .60. However, according the AVE, only the four factors *Hospital management support for patient safety, Hospital handoffs and transitions, Feedback and communication about error *and *Frequency of event reporting *reached acceptable values of ≥ .50.

**Table 4:** Local fit of items within the 12 HSOPS_M dimensions

Dimension	Item	Indicator reliability	Factor reliability	AVE	FLR
***Safety Culture Dimensions***					

Hospital management support for patient safety	F1	.663	.87	.70	.91
	F8	.704			
	F9	.710			

Supervisor/manager expectations/actions	B1	.270	.71	.39	1.72
	B2	.463			
	B3	.261			
	B4	.590			

Teamwork across hospital units	F2	.413	.79	.48	1.34
	F4	.567			
	F6	.350			
	F10	.654			

Teamwork within units	A1	.465	.78	.47	1.25
	A3	.552			
	A4	.606			
	A11	.305			

Communication openness	C2	.519	.65	.38	2.00
	C4	.260			
	C6	.376			

Hospital handoffs and transitions	F3	.685	.82	.54	1.21
	F5	.444			
	F7	.664			
	F11	.405			

Nonpunitive response to error	A8	.410	.73	.48	1.42
	A12	.636			
	A16	.383			

Feedback and communication about error	C1	.564	.79	.56	1.37
	C3	.564			
	C5	.556			

Staffing	A2	.504	.75	.43	.93
	A5	.398			
	A7	.193			
	A14	.631			

Organizational learning	A6	.361	.62	.36	1.94
	A9	.341			
	A13	.365			

***Outcome Dimensions***					

Overall perceptions of safety	A10	.452	.73	.40	1.72
	A15	.299			
	A17	.431			
	A18	.475			

Frequency of event reporting	D1	.746	.87	.69	0.55
	D2	.707			
	D3	.593			

### Construct validity

Concerning the FLR (Table 4), only three dimensions (*Hospital management support for patient safety, Staffing *and *Frequency of event reporting*) showed acceptable values. All of the other values indicated that these dimensions were not sufficiently distinguishable from the other dimensions within the model. The inter-correlations for the 12 safety culture dimensions are shown in Table 5. The correlations ranged from .13 (between *Staffing *and *Frequency of event reporting*) to .64 (between *Hospital management support *and *Overall perception of safety*). Except for the correlation between *Staffing *and *Frequency of event reporting, a*ll correlations reached acceptable values between .20 and .70. Nonetheless, several intercorrelations reached values higher .5. This indicated that there was not at all a moderate relationship between the safety culture dimensions, and supported the result, that not all safety culture dimensions were sufficiently distinguishable. Furthermore, we calculated correlations between the 12 safety culture dimensions and the individual outcome measure *Patient safety grad*e. The lowest correlation of this outcome measure was with *Staffing *(r = 0.32), and the highest correlation was with *Overall perceptions of safety *(r = 0.62).

**Table 5:** Inter-correlations of the 12 HSOPS_M dimensions

Factor	PSG	1	2	3	4	5	6	7	8	9	10	11
1 Hospital management support for patient safety	.50											
2 Supervisor/manager expectations/actions	.48	.60										
3 Teamwork across hospital units	.49	.58	.55									
4 Teamwork within units	.45	.47	.49	.59								
5 Communication openness	.37	.47	.52	.46	.44							
6 Hospital handoffs and transitions	.49	.53	.48	.62	.46	.40						
7 Nonpunitive response to error	.43	.50	.54	.53	.46	.48	.49					
8 Feedback and communication about error	.38	.60	.54	.48	.47	.62	.43	.47				
9 Staffing	.32	.44	.38	.32	.34	.22	.42	.38	.27			
10 Organizational learning	.44	.59	.53	.49	.55	.50	.41	.43	.59	.22		
11 Overall perceptions of safety	.62	.64	.60	.54	.48	.46	.57	.60	.53	.48	.56	
12 Frequency of event reporting	.33	.42	.39	.32	.30	.47	.33	.32	.49	.13	.45	.41

*NOTE: *PSG = Patient Safety Grade. All correlations are significant at *p *≤ 0.001.

### Reliability

The reliability measured by Cronbach's alpha ranged from .61 to .87 (Table 6), whereas the levels of Cronbach's alphas for *Supervisor/manager expectations/actions, Communication openness *and *Organizational learning *were below an adequate value of .70. The other dimensions reached acceptable reliability coefficients. Compared with the results found from the US data, *Staffing *had a much higher alpha in the German data. However, the scales *Communication openness *and *Organizational learning *had much lower alphas in the German data.

**Table 6:** Reliability of the 12 safety culture dimensions in the German data compared with the US data

Dimension	No. of items	Cronbach's alpha American data for staff^a^	Cronbach's alpha German data for management
***Safety Culture Dimensions***			
Hospital management support for patient safety	3	.83	.87
Supervisor/manager expectations/actions	4	.75	.69
Teamwork across hospital units	4	.80	.78
Teamwork within units	4	.83	.77
Communication openness	3	.72	.64
Hospital handoffs and transitions	4	.80	.83
Nonpunitive response to error	4	.79	.73
Feedback and communication about error	3	.78	.79
Staffing	4	.63	.73
Organizational learning	3	.76	.61
***Outcome Dimensions***			
Overall perceptions of safety	4	.74	.73
Frequency of event reporting	3	.84	.86

*NOTE: **^a ^***Results of the pilot study [15]

## Discussion

The HSOPS is one of the most frequently used questionnaires to assess safety culture in health care settings. Until now, this questionnaire has been used to evaluate safety culture from employees' points of view. There exist an increasing number of studies testing how consistently the HSOPS questionnaire measures safety culture dimensions [18,29,31-33]. However, these surveys have all been tested with medical staff only. Because it is important to test whether the HSOPS is also applicable for assessing single views of a hospital's safety culture, the purpose of our study was to test the psychometric properties of the HSOPS adapted for hospital management (HSOPS_M).

The CFA indicated that the factor structure of the original HSOPS fits the data of the German version for medical directors. The factor model exhibited an acceptable-to-good global data fit. Furthermore, the local fit indices were considered acceptable. Regarding the indicator reliability, most indicators, except one, exceeded the acceptable values. All factors reached the recommended critical values for the factor reliabilities, but only four factors reached adequate AVE values. These results suggested a good convergent validity. The results of the local fit indicators, especially for the AVE, found in this study are comparable to the results of the Swiss-German version of the HSOPS [18].

According the construct validity, only three dimensions reached satisfactory values for the FLR. Therefore, the construct validity of the factor model can be considered less acceptable. The values of FLR indicated that the factors measured not readily distinguishable dimensions. The values of the FLR are similar to the Swiss data analysis [18]. Furthermore, the inter-correlations between all 12 safety culture dimensions ranged between .13 and .64. We found several correlations higher than .5, which supported the result that the dimensions were not at all independent of each other. One possible reason for the high values of FLR and high intercorrelations could be that theoretically correlated dimensions (e.g. *Feedback and communication about error *and *Communication openness; Hospital management support for patient safety *and *Supervisor/manager expectations/actions*) are measured with different constructs. This suggested further investigation, especially on the question whether these dimensions should be measured in one dimension. Nonetheless, as expected, all 12 safety culture dimensions correlated with the outcome variable *Patient safety grade*. We found a high correlation between *Patient safety grade *and *Overall perceptions of safety*, which is a good indication of the validity of the latter dimension.

Finally, the analysis of Cronbach's alpha signified that the dimensions have an acceptable level of reliability. In nine out of the 12 dimensions hypothesised in the origin factor model, the Cronbach's alpha ranged between .73 and .87. In addition, the alpha of the factor *Supervisor/manager expectations/actions *was not much below the recommended cut-off value of .70. In particular, for *Communication openness *and *Organizational learning*, the lower values of Cronbach's alpha can probably be attributed to the different survey designs (e.g. measuring management perception versus the perceptions of frontline staff). Nevertheless, a comparison of these reliabilities with other European HSOPS surveys showed that *Communication openness *[18,32] and *Organizational learning *[18,31,32] repeatedly had low Cronbach's alpha values.

Overall, the construct validity indicated that further scale refinement is needed to improve the questionnaire. To minimise differences between the survey versions, we refrained from reducing or adding any scales within the instrument. Nonetheless, model modifications should not generally be excluded. Especially in cases of high intercorrelations between dimensions, which are theoretically high correlated, further scale refinement could lead to better psychometric properties. In this respect, we agree with Pfeiffer and Manser [18] that the set of dimensions within the HSOPS still has to be optimised.

The findings of our study are limited by the following aspects. The results of this study are based on a cross-sectional mail survey with a response rate of 45%. Although little is known about potential non-response bias with these kinds of surveys, we assumed that the attitudes of the responding medical directors do not differ from those of non-responding medical directors [34].

Within the scope of this study, we were not able to examine the relationship between patient safety culture and objective patient safety outcomes, such as patient safety indicators or frequencies of medical errors. Therefore, we agree with previous suggestions [7,29] that more evidence is needed on the relationship between patient safety culture and patient safety outcomes.

Comparing the psychometric properties of the HSOPS_M to those of the original HSOPS for hospital employees means not only comparing different countries, but also different methods. Most safety culture surveys are used to measure safety culture from the frontline staff's points of view. Assessing safety culture only with medical directors excludes the views of frontline staff and does not take the potential differences between hospital units [35-38] into account. Therefore, we think the area of application of the HSOPS_M is different from traditional hospital-related safety culture instruments. According to Rousseau [14], we presupposed that the points of view of key informants, such as medical directors, were representative of hospital professionals in identifying safety culture for the whole hospital. Hospital managers are expected to make decisions regarding quality improvement and patient safety issues. In addition, essential decision makers - such as medical directors - have a comprehensive knowledge about their organisations. Therefore, questioning the top management offers a different approach to measuring safety culture and providing aggregated organisational data. For analysing the safety culture in different hospitals units, further research should consider using the HSOPS_M for hospital unit managers as well.

Finally, the HSOPS_M was embedded in a larger questionnaire within the HOSQua-study, which could be a possible factor that influenced the responses of the medical directors. According to Linsky [39], we assumed that the length of the questionnaire would not necessarily influence the validity or reliability of the HSOPS. Nonetheless, further analyses on validity and reliability should be performed using the HSOPS_M questionnaire only.

## Conclusion

The HSOPS questionnaire covers a wide range of sub-dimensions. In addition to the most frequently included concepts, such as management commitment, supervisor commitment, communication openness, and safety system, the HSOPS uses dimensions such as feedback and communication about errors/events, organizational learning, handoffs and transitions, staffing and teamwork. As such, the HSOPS provides a broad range of important information on safety culture, although, the results of the psychometric properties of the HSOPS_M suggest that further scale refinement and model modifications are needed. While the lack of confirmation of factor structure was found in other European studies using the HSOPS for hospital employees, it should be noted that several dimensions emerged relatively consistently across national settings. Therefore, we suggest investigating the HSOPS_M in different national and international settings, to optimise the set of safety culture dimensions. The HSOPS_M could then be used in nationwide hospital surveys to assess the top management's views on safety culture in hospitals for subsequent inter-organisational comparisons. For example, the HSOPS_M could be used as a measurement to prove interventions on safety performance in hospitals from the top management's points of view. Nonetheless, further research regarding the relationship between safety culture and other variables (e.g., mortality rates and patient safety indicators) is needed.

## List of abbreviations used

(AVE): Average Variance Extracted; (Chi^2^/df): Chi-squared value; (CFI): Comparative Fit Index; (CFA): Confirmatory factor analysis; (HOSQua): Effects of Hospital Ownership Structures on Quality of Health Care; (EM): Expectation maximization; (FLR): Fornell-Larcker Ratio; (HSOPS): Hospital Survey on Patient Safety Culture; (HSOPS_M): Hospital Survey on Patient Safety Culture for hospital management; (IOM): Institute of Medicine; (KMO): Kaiser-Meyer-Olkin coefficient; (MSA): Measure of Sample Adequacy coefficient; (RMSEA): Root Mean Square Error of Approximation; (SRMR): Standardised Root Mean Residual; (TLI): Tucker-Lewis Index.

## Competing interests

The authors declare that they have no competing interests.

## Authors' contributions

AH - Conception and design of the study, data gathering, data analysis, interpretation of the data, drafting of the manuscript, revising manuscript, final approval; NE - Interpretation of the data, revising manuscript, interpretation of the data, final approval; OO - Operative project leader, conception and design of the study, revising manuscript, final approval; MW - Data analysis, revising manuscript, interpretation of the data, final approval; TM, YP - Support in adapting the HSOPS for medical directors, editing the retranslation, revising manuscript, final approval; HP - Conception and design of the study, revising manuscript, interpretation of the data, final approval

## Pre-publication history

The pre-publication history for this paper can be accessed here:

http://www.biomedcentral.com/1472-6963/11/165/prepub

## Supplementary Material

Additional file 1 **Hammer_BMC_HSOPS_M_Questionnaire**. Hospital Survey on Patient Safety Culture for hospital management (HSOPS_M). HSOPS_M (English version) Click here for file
