# Supplementary material for: Psychometric properties of the Hospital Survey on Patient Safety Culture for hospital management (HSOPS_M)
Source: BMC Health Serv Res. 2011 Jul 11;11:165. doi: 10.1186/1472-6963-11-165 (PMC3148962; doi:10.1186/1472-6963-11-165)
Supplement: Additional file 1 — Hammer_BMC_HSOPS_M_Questionnaire. Hospital Survey on Patient Safety Culture for hospital management (HSOPS_M). HSOPS_M (English version) [file 1472-6963-11-165-S1.PDF]

Hospital Survey on Patient Safety Culture for hospital management (HSOPS_M)

1.	Please indicate your level of agreement with the following statements about the average units in this hospital. Mark your answer by placing an “X” in the appropriate box.	Strongly Disagree	Disagree	Neither	Agree	Strongly Agree
A1	Staff support one another within the units.	☐	☐	☐	☐	☐
A2	Units within this hospital have enough staff to handle the workload.	☐	☐	☐	☐	☐
A3	When a lot of work needs to be done quickly, staff within the units work together as a team to get the work done.	☐	☐	☐	☐	☐
A4	Staff within the units treat each other with respect.	☐	☐	☐	☐	☐
A5r	Unit staff work longer hours than is best for patient care.	☐	☐	☐	☐	☐
A6	Staff within the units are actively doing things to improve patient safety.	☐	☐	☐	☐	☐
**2.**	**Please indicate your level of agreement with the following statements about the average units in this hospital. Mark your answer by placing an “X” in the appropriate box.**	**Strongly Disagree**	**Disagree**	**Neither**	**Agree**	**Strongly Agree**
A7r	The units use more agency / temporary staff than is best for patient care.	☐	☐	☐	☐	☐
A8r	Staff within the individual units feel like their mistakes are held against them.	☐	☐	☐	☐	☐
A9	Mistakes have led to positive changes within the hospital units.	☐	☐	☐	☐	☐
A10r	It is just by chance that more serious mistakes don’t happen within the units.	☐	☐	☐	☐	☐
A11	When one area within a unit gets really busy, others help out.	☐	☐	☐	☐	☐
A12r	When an event (e.g., mistake) is reported, it feels like the person is being written up, not the problem.	☐	☐	☐	☐	☐
**3.**	**Hospital Units: Please think about the average units in this hospital.**	**Strongly Disagree**	**Disagree**	**Neither**	**Agree**	**Strongly Agree**
A13	After changes have been made to improve patient safety within the units, their effectiveness is evaluated by the staff.	☐	☐	☐	☐	☐
A14r	Staff within units work in “crisis mode” trying to do too much, too quickly.	☐	☐	☐	☐	☐
A15	Patient safety is never sacrificed to get more work done.	☐	☐	☐	☐	☐
A16r	Staff worry that mistakes they make are kept in their personnel file.	☐	☐	☐	☐	☐
A17r	We have patient safety problems within the units.	☐	☐	☐	☐	☐
A18	Unit procedures and systems are good at preventing errors from happening.	☐	☐	☐	☐	☐

4.	Please give for all the units in this hospital an overall grade on patient safety.				
E1	**Excellent** ☐	**Very Good** ☐	**Acceptable** ☐	**Poor** ☐	**Failing** ☐

5.	Supervisors / Managers: Please indicate your level of agreement with the following statements about the supervisors / managers (at all hierarchical levels) in this hospital.	Strongly Disagree	Disagree	Neither	Agree	Strongly Agree
B1	Supervisors / managers say a good word when they see that a job has been done according to established procedures (standards and guidelines).	☐	☐	☐	☐	☐
B2	Supervisors / managers seriously consider staff suggestions for improving patient safety.	☐	☐	☐	☐	☐
B3r	Whenever pressure builds up, supervisors / managers want staff to work faster, even if it means taking shortcuts or skipping steps.	☐	☐	☐	☐	☐
B4r	Supervisors / managers overlook patient safety problems that happen over and over.	☐	☐	☐	☐	☐

6	**Communications**<br>**Please think about the average units in this hospital.**	**Never**	**Rarely**	**Some-times**	**Most of the time**	**Always**
C1	Staff within units are given feedback about changes put into place based on events reported (e.g., mistakes).	☐	☐	☐	☐	☐
C2	Staff within units will freely speak up if they see something that may negatively affect patient care.	☐	☐	☐	☐	☐
C3	Staff within units are informed about events (e.g., errors) that happen in their units.	☐	☐	☐	☐	☐
C4	Staff within units feel free to question the decisions or actions of those with more authority.	☐	☐	☐	☐	☐
C5	Staff within units discuss ways to prevent an event (e.g., error) from happening again.	☐	☐	☐	☐	☐
C6r	Staff within units are afraid to ask questions when something does not seem right.	☐	☐	☐	☐	☐
**7.**	**Event Reporting:**<br>**When the following events (e.g., errors) occur within the hospital units, how often do you think they are reported?**	**Never**	**Rarely**	**Some-times**	**Most of the time**	**Always**
D1r	When an event (e.g., error) occurs <u>that is caught and corrected before affecting the patient</u>, how often is this reported?	☐	☐	☐	☐	☐
D2r	When an event (e.g., error) occurs <u>that poses no potential harm to the patient</u>, how often is this reported?	☐	☐	☐	☐	☐
D3r	When an event (e.g., error) occurs <u>that could harm the patient, but does not</u>, how often is this reported?	☐	☐	☐	☐	☐
**8.**	**The Hospital:**<br>**Please indicate your level of agreement with the following statements about your hospital.**<br>**Please think about your hospital as a whole.**	**Strongly Disagree**	**Disagree**	**Neither**	**Agree**	**Strongly Agree**
F1	Hospital management provides a work climate that promotes patient safety.	☐	☐	☐	☐	☐
F2r	Hospital units do not coordinate well with each other.	☐	☐	☐	☐	☐
F3r	Things “fall between the cracks” when transferring patients from one unit to another.	☐	☐	☐	☐	☐
F4	There is good cooperation among hospital units that need to work together.	☐	☐	☐	☐	☐
F5r	Important patient care information is often lost during shift changes within the hospital units.	☐	☐	☐	☐	☐
F6r	It is often unpleasant for staff from one hospital unit to work with staff from other hospital units.	☐	☐	☐	☐	☐
F7r	Problems often occur during the exchange of information across hospital units.	☐	☐	☐	☐	☐
F8	The actions of hospital management show that patient safety is a top priority.	☐	☐	☐	☐	☐
F9r	Hospital management seems to be interested in patient safety only after an adverse event happens.	☐	☐	☐	☐	☐
F10	Hospital units work well together to provide the best care for patients.	☐	☐	☐	☐	☐
F11r	Shift changes are problematic for patients within the hospital units.	☐	☐	☐	☐	☐
